# Sustained Accumulation of Molecular Clock Suppressors Period 1 and Period 2 Promotes C2C12 Myotube Atrophy Through an Autocrine-Mediated Mechanism With Relevance to Androgen Deprivation-Induced Limb Muscle Mass Loss

**DOI:** 10.1093/function/zqaf030

**Published:** 2025-07-09

**Authors:** Grant R Laskin, Jennifer L Steiner, Wayne A Ayers-Creech, Michael L Rossetti, Kirsten R Dunlap, Cynthia Vied, Choogon Lee, Nicholas P Greene, Dennis K Fix, Orlando Laitano, Kislay Parvatiyar, Bradley S Gordon

**Affiliations:** Department of Health, Nutrition, and Food Sciences, Florida State University, Tallahassee, FL 32306, USA; Department of Health, Nutrition, and Food Sciences, Florida State University, Tallahassee, FL 32306, USA; Institute of Sports Sciences and Medicine, Florida State University, Tallahassee, FL 32306, USA; Department of Health, Nutrition, and Food Sciences, Florida State University, Tallahassee, FL 32306, USA; Department of Health, Nutrition, and Food Sciences, Florida State University, Tallahassee, FL 32306, USA; Department of Health, Nutrition, and Food Sciences, Florida State University, Tallahassee, FL 32306, USA; Translational Science Laboratory, Florida State University College of Medicine, Tallahassee, FL 32306, USA; Department of Biomedical Sciences, Florida State University College of Medicine, 115 W Call Street, Tallahassee, FL 32304, USA; Department of Health, Human Performance and Recreation, Cachexia Research Laboratory, Exercise Science Research Center, University of Arkansas, Fayetteville, AR 72701, USA; Recursion Pharmaceuticals, Salt Lake City, UT 84101, USA; Department of Applied Physiology and Kinesiology, University of Florida, Gainesville, FL 32611, USA; Department of Health, Nutrition, and Food Sciences, Florida State University, Tallahassee, FL 32306, USA; Department of Health, Nutrition, and Food Sciences, Florida State University, Tallahassee, FL 32306, USA; Institute of Sports Sciences and Medicine, Florida State University, Tallahassee, FL 32306, USA

**Keywords:** inflammation, hypogonadism, RNA sequencing, circadian rhythm

## Abstract

Low testosterone in males (hypogonadism) is associated with limb muscle mass loss, yet the underlying mechanisms of muscle mass loss remain largely unknown. We previously showed androgen deprivation disrupted limb muscle molecular clock function, and the disruption coincided with elevated levels of the primary molecular clock suppressor, Period 2 (*Per2*). The purposes herein were to determine if PER2 overexpression leads to muscle atrophy and if preventing PER2 accumulation blunts limb muscle mass loss in response to androgen deprivation. Here, we identify *Per2* as a negative regulator of muscle size. Overexpression of *Per2* in differentiated C2C12 myotubes reduced myotube diameter, while deletion of *Per2* in male mice partially preserved tibialis anterior (TA) mass following castration. The muscle-sparing effect of *Per2* deletion in vivo was specific to the TA despite evidence of molecular clock disruption and mass loss in other muscles. Subsequently, we show overexpression of the other primary clock suppressor, Period 1 (*Per1*) also reduced myotube diameter in differentiated C2C12 myotubes. Mechanistically, both *Per1* and *Per2* overexpression *in vitro* induced muscle atrophy in part by an autocrine-mediated mechanism likely involving inflammation as their overexpression induced an inflammatory gene expression signature and increased cytokine/chemokine secretion. Moreover, incubation of C2C12 myotubes in the media conditioned from *Per1* or *Per2* overexpressing myotubes reduced myotube diameter. Several inflammatory genes identified *in vitro* were also altered in the limb muscles in response to androgen deprivation. These findings identify a previously unrecognized role for *Per1/2* in regulating skeletal muscle mass with implications for muscle loss during hypogonadism.

## Graphical Abstract

**Graphical Abstract:**
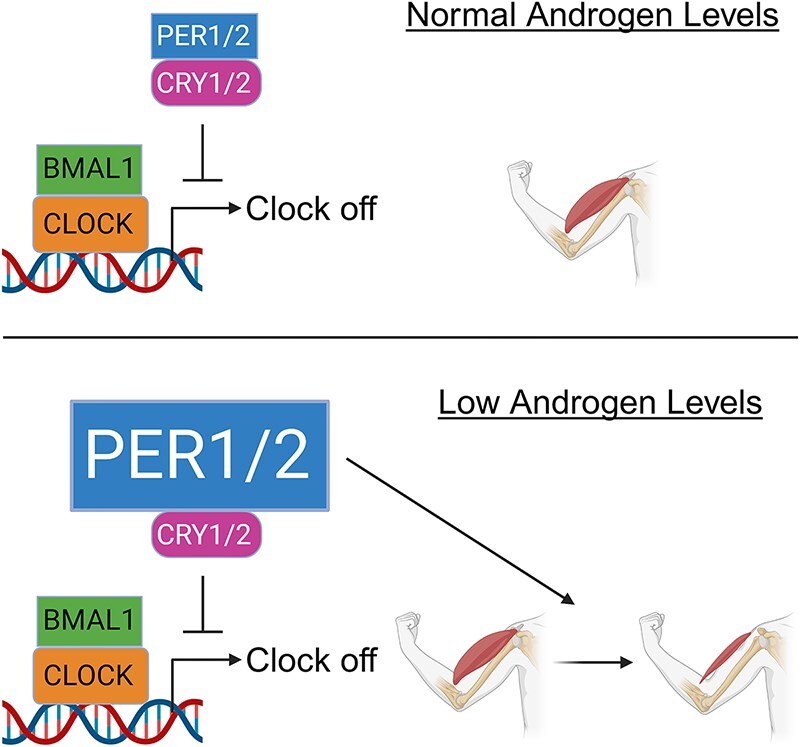

## Introduction

Skeletal muscle is the largest organ in the body by mass with important functions related to locomotion and whole-body metabolic homeostasis.^1^ Limb muscles account for the majority of total muscle mass,^2^ meaning they are central to movement and metabolic homeostasis. Low testosterone levels in males (hypogonadism) are strongly associated with the loss of limb muscle mass, and restoring testosterone levels to the normal physiological range in hypogonadal men can increase limb muscle mass.^3^^,^^4^ However, testosterone treatment may not be well tolerated by those hypogonadal men that could benefit from it the most. For instance, a study testing the efficacy of testosterone replacement in frail, elderly hypogonadal men was stopped early due to greater incidence of adverse cardiovascular events in the testosterone treated group.^5^ Additionally, testosterone enhances malignant tumor growth,^6^^,^^7^ posing a significant risk for cachectic hypogonadal men with malignant tumors. Safe and effective alternatives are needed, but their development is hindered because the molecular mechanisms driving limb muscle mass loss during hypogonadism remain largely unknown.

The presumed pathway by which testosterone regulates muscle mass is via the androgen receptor. However, genetic deletion of the androgen receptor in vivo does not induce limb muscle mass loss^8^^,^^9^^,^^10^^,^^11^ indicating it is the presence of androgens, not androgen receptor activity, that regulates limb muscle mass.^11^ Other proposed androgen-sensitive factors have also not proven functional in vivo. For example, treating myotubes in culture with supraphysiological levels of testosterone increased myotube diameter in a manner that appears to be fully dependent upon the mechanistic target of rapamycin in complex 1 (mTORC1) pathway.^12^ However, our in vivo work showed inhibiting mTORC1 in castrated rodents did not prevent testosterone from fully restoring limb muscle mass to uncastrated levels.^13^ Thus, the molecular factors by which androgens have been proposed to control muscle mass have not proven effective at regulating limb muscle mass in vivo.

Our laboratory’s search for pathways altered in the limb muscle following androgen deprivation identified disruptions to the limb muscle molecular clock.^14^ The molecular clock is a transcription-translation feedback loop that has emerged as an important regulator of skeletal muscle homeostasis.^15^^,^^16^ The clock consists of a positive arm comprised of the transcription factors Brain and Muscle ARNT-Like 1 (BMAL1) and Circadian Locomoter Output Cycles Protein Kaput (CLOCK). The negative arm of the clock is comprised of the Period (PER) and Cryptochrome (CRYs) proteins.^17^^,^^18^^,^^16^ While three PER proteins have been identified (PER1-3), PER1 and PER2 are considered the primary components of the negative arm of the clock as they dimerize with the CRYs to suppress the transcriptional activity of the BMAL1/CLOCK complex.^17^

Turnover of the PER1/2 proteins is the primary node regulating clock function as sustained accumulation of either PER1 or PER2 strongly disrupts clock function.^17^^,^^18^^,^^19^ Accordingly, our laboratory showed molecular clock disruption in the limb muscles coincided with elevated PER2 protein content (PER1 was not assessed in that report),^14^ suggesting the molecular clock disruption was due at least in part to sustained accumulation of PER2. While molecular clock disruption does not appear to directly regulate limb muscle mass per se as *Bmal1* deletion in developed muscle does not promote atrophy,^20^^,^^16^ it is possible sustained PER2 accumulation may yield muscle atrophy by functioning outside of the clock. For instance, high levels of PER2 in cancer cells exhibit functions outside of the core clock including activation of pathways such as p53,^21^ which is known to promote muscle atrophy and is hyperactive in the limb muscle when androgen levels are low.^22^^,^^23^ Therefore, we tested the hypotheses that PER2 overexpression is sufficient to induce muscle atrophy and preventing PER2 accumulation blunts the loss of limb muscle mass in response to androgen deprivation. Herein, we show *Per2* overexpression induces muscle atrophy in vitro, and *Per2* deletion in male mice partially preserves tibialis anterior (TA) mass in response to androgen deprivation. Mass of other limb muscles following androgen deprivation were not affected by *Per2* deletion despite evidence of clock disruption and mass loss in those muscles,^14^ prompting us to test the hypothesis that *Per1* overexpression also induces muscle atrophy. We demonstrate *Per1* overexpression also yields muscle atrophy in vitro. Together, these findings support a model whereby PER1 and/or PER2 accumulation contribute to limb muscle mass loss when androgen levels are low.

## Materials and Methods

### C2C12 Cell Culture, Adenovirus Transduction, and Myotube Diameter

C2C12 cell culture experiments were conducted as previously described.^24^ C2C12 myoblasts were purchased from ATCC (cat. #CRL-1772; Manassas, VA, USA) and cultured subconfluently at 37°C in 5% CO_2_ in complete growth media consisting of high glucose Dulbecco’s modified Eagle’s Media (DMEM; cat. #10-017-CV; Corning Life Sciences, Tewksbury, MA, USA) containing 10% fetal bovine serum (FBS; cat. #EF-0500-A; Atlas Biologicals, Fort Collins, CO) and 1% penicillin-streptomycin (PS; cat. #15140122; Thermo Fisher Scientific, Waltham, MA, USA). Experiments were conducted in 12- or 24-well plates (cat. #10062-894 or 10062-896; VWR, Radnor, PA, USA) pre-coated with a Type I rat tail collagen matrix. Coating consisted of diluting RatCol collagen solution (cat. #5056-20ML; Advanced Biomatrix, Carlsbad, CA, USA) and acetic acid into sterile water to a final concentration of 100 µg/mL for rat collagen and 0.1% acetic acid. Five hundred µL of the final solution was added to each well of the plate and incubated under UV light for 2 h to allow collagen to polymerize. Prior to plating myoblasts, the collagen solution was aspirated, and wells were rinsed twice with sterile phosphate buffered saline. Myotube differentiation was initiated when cells reached ∼95-100% confluence by changing to differentiation media consisting of high glucose DMEM containing 2% horse serum (HS; cat. #16050130; Thermo Fisher Scientific, Waltham, MA, USA), 1% PS, and 1 µm insulin (cat. #11070-73-8; Sigma-Aldrich, Burlington, MA, USA).

C2C12 myoblasts were differentiated for 3 days prior to experimentation. Differentiation was confirmed prior to experimentation on day 4 by the presence of multinucleated mature myotubes covering the wells. After confirmation, C2C12 myotubes were transduced using adenoviruses obtained from Vector Biolabs (Malvern, PA, USA) encoding *Per1 + Gfp* (Ad-*Gfp*-m-*Per1*;), *Per2 + Gfp* (Ad-*Gfp*-m-*Per2*), or adenovirus encoding *Gfp* only as a control (Ad-CMV-*Gfp*; cat. #1060). Adenoviruses encoding *Per1* (RefSeq#: BC091645) and *Per2* (RefSeq#: NM_011066) were custom made by Vector Biolabs. The expression of all genes was driven by cytomegalovirus (CMV) promoters with *Per1/2* and *Gfp* driven by separate CMV promoters within the same viral vector. Transduction consisted of adding the adenovirus directly to the differentiation media at final viral concentrations of 3.2 × 10^8^ PFU/mL of media (*Per1 *+ *Gfp* and *Per2* + *Gfp)*, and 8.0 × 10^7^ PFU/mL of media for the *Gfp* only vectors. The viral concentrations used in the experiments were those which produced the highest transduction efficiency (eg, highest number of GFP-positive myotubes) while maintaining cell viability. Twenty-four hours after transduction, virus-containing media was changed to fresh differentiation media for experiments lasting 48 h. Twenty-four- or fourty-eight-h post-transduction, nonoverlapping myotube images from the entire well were acquired at 200× on a Leica DMi8 microscope (Wetzler, Germany) mounted with a Leica DFC7000 T camera using Leica Application Suite X software. Bright field and fluorescent images were taken concurrently. To circumvent fluorescent interference from cellular debris and/or unfused myoblasts, myotube diameter was measured on the bright field image with the corresponding fluorescent image used to determine GFP-positive myotubes for measurement. Only GFP-positive myotubes with an elongated, mature, non-nascent morphology were analyzed (visualization is found in^25^).

The diameters of ≥72 myotubes per transduction condition were determined in each experimental replicate as follows. For each myotube, 5 diameter measurements were taken along its length and averaged to obtain a single diameter. These individual myotube diameters were then averaged to yield a single diameter value per transduction condition. Mean diameters of *Per1* or *Per2* transduced myotubes were normalized to the mean diameter of *Gfp* transduced myotubes within the same replicate. Normalized values from the three independent replicates were averaged for final analysis. All measurements were performed using ImageJ software (NIH, Bethesda, MD, USA) by an investigator blinded to the transduction conditions. Each replicate included *Gfp* and *Per* transduction conditions.

Separate C2C12 myotube experiments were used for gene expression analysis or protein analysis and each consisted of 3 independent experimental replicates, with each experimental replicate containing between 2 to 6 biological replicates per virus. For RNA analysis, cells were harvested in 500 μL TRI Reagent (Zymo Research, Irvine, CA, USA). Gene expression was assessed relative to the *Gfp* only condition within each experimental replicate, and the gene expression fold changes from each experimental replicate were averaged to generate the final dataset. For gene expression experiments, we transduced more wells with *Per1* and *Per2* than *Gfp* within each experimental replicate because of a limited quantity of *Gfp* adenovirus. No samples/wells were removed due to outlying values in gene expression. For protein analysis, transduced myotubes were harvested in buffer containing 50 mm Hepes (pH 7.4), 0.1% Triton X-100, 4 mm EGTA, 10 mm EDTA, 50 mm Na_4_P_2_O_7_, 100 mm β-glycerophosphate, 25 mm NaF, 5 mm Na_3_VO_4_, and 10 μL/mL of protease inhibitor cocktail (cat. #*P*-8340; Sigma-Aldrich). The protein content of each sample was quantified via the Bradford method, and equal quantities of protein were diluted in 2X Laemmle buffer.

### Conditioned Media and Myotube Diameter

C2C12 myoblasts were differentiated in 12-well plates and transduced with adenovirus as described above (one plate for each virus). Twenty-four hours post-transduction, virus containing media was replaced with fresh differentiation media. Forty-eight hours post-transduction, media from each transduction condition was harvested (*n* = 5 wells per transduction condition), centrifuged at 1000 *g* for 10 min at 4°C, and frozen and stored at −80°C. Those conditioned media replicates (*n* = 5/transduction condition) were subjected to multiplex analysis described below. The remaining media from each plate/transduction condition was collected and pooled. The pooled conditioned media from each transduction was centrifuged at 1000 *g* for 10 min to pellet any cellular debris.

The debris-free conditioned media was immediately added to fully differentiated C2C12 myotubes or frozen and stored at −80°C for subsequent use. For each subsequent use, the frozen media was thawed in a 37°C bead bath immediately before adding the media to the myotubes. Myotubes were incubated in the conditioned media for 24 h before assessing myotube diameter using the same procedures described in *Adenovirus transduction and myotube diameter*. Alternatively, myotubes were incubated in conditioned media for 1 h prior to harvest in 1× Laemmli sample buffer before assessing protein signaling by Western blot analysis.

### Animals

Male C57Bl/6 J mice were purchased from Jackson Laboratories (Bar Harbor, ME, USA). Male C57Bl/6 mice with the *Per2* gene disrupted (*Per2*−/−) were provided by Dr. Choogon Lee and bred with C57Bl/6 J mice to generate *Per2 *+/- mice. *Per2 *+/- mice were bred together to generate the *Per2*−/− mice and corresponding *Per2*+/+ littermates. All mice were housed in a temperature- (25°C) and light- (12 h light/12 h dark) controlled environment within the vivarium at Florida State University (FSU). Mice were provided standard 5001 rodent chow (LabDiet, St. Louis, MO, USA) and access to water *ad libitum* throughout the studies. Animals were euthanized by cardiectomy while under deep isoflurane anesthesia. All animal facilities and experimental procedures were approved by the Institutional Animal Care and Use Committees of FSU.

### Castration of *Per2*+/+ and *Per2*−/− Mice

At 16 wk of age, male *Per2*+/+ and male whole body *Per2*−/− mice were each randomized into 2 groups of equal body weight. One group of mice from each genotype was subjected to castration surgery where the testicles were removed through a ∼1 cm incision in the lower abdomen. The other groups from each genotype were subjected to sham surgery that included the same procedures, but the testicles were left intact. All surgical procedures were performed under deep isoflurane anesthesia (3%) and post-operative pain was alleviated with 2 subcutaneous injections of buprenorphine (0.1 mg/kg/injection), with each injection separated by 5 h. The castration procedure has been previously described.^13^ All mice recovered for 8 wk to ensure development of limb muscle mass loss. Body composition was then assessed by EchoMRI and tissues were harvested, frozen in liquid nitrogen, and stored at −80°C.

### Androgen Deprivation Time Course

Fourteen-week-old male mice were randomized into eleven groups of equal body mass. One group was euthanized on the day of the surgery to establish baseline body/organ/tissue phenotype values (*N* = 4). Five of the remaining groups were subjected to castration surgery (*N* = 3/group) while the other 5 remaining groups were subjected to sham surgery (*N* = 3/group) as described above. A group of mice subjected to castration or sham surgeries were each euthanized at 7, 14-, 21-, 28-, and 49-days post-surgery, which represents the time course of limb muscle mass loss. All tissues were harvested under deep isoflurane anesthesia (3%). To minimize influence of diurnal variation on our outcome measures, tissues from sham and castrated mice were collected at the same time on each day of harvest throughout the time course, with all days of harvest occurring ∼5-8 h into the dark cycle. All samples were collected in the freely fed metabolic state. Following extraction, all tissues were flash frozen in liquid nitrogen and stored at −80°C until further analysis.

### RNA Extraction, cDNA Synthesis, and Quantitative Real Time Polymerase Chain Reaction (qRT-PCR)

RNA from ∼20 mg of TA or gastrocnemius muscle was homogenized in 600 µL of Zymo Tri Reagent (Irvine, CA, USA). The RNA from homogenized muscle or C2C12 myotubes was isolated using a Zymo Direct-zol RNA Miniprep kit with on-column DNase treatment (Irvine, CA, USA; cat. #R2050). RNA quantity and purity was determined spectrophotometrically by the 260- to 280-nm ratio. cDNA was synthesized from 0.75-1 µg of RNA using a High-Capacity cDNA Reverse Transcription Kit (Thermo Fisher Scientific, Waltham, MA, USA; cat. #4368814). qRT-PCR was conducted on either QuantStudio3 (Thermo Fisher Scientific, Waltham, MA, USA) or QuantStudio7 (ThermoFisher Scientific) using PowerUp SYBR Green Master Mix (cat. #A25742; Thermo Fisher Scientific) or TaqMan Fast Advanced Master Mix (cat. #4444557; Applied Biosystems, Foster City, CA, USA). The conditions for qRT-PCR with SYBR Green were an initial 2 min at 50°C and 2 min at 95°C, followed by 40 cycles with each cycle consisting of a 15 s denature step at 95°C, a 15 s annealing step at 55°C, and a 1-min extension step at 72°C. A melt curve analysis was performed for each primer pair to ensure that a single product was amplified, and the product sizes for each primer pair were verified via agarose gel electrophoresis prior to experimentation. The conditions for TaqMan were an initial 2 min at 50°C and 10 min at 95°C, followed by 45 cycles with each cycle consisting of a 15 s denature step at 95°C, a 1 min annealing step at 60°C, and a 1-min extension step at 60°C. Relative expression levels of each target gene were normalized using the ΔΔC_t_ method with *Rplp0* as the reference gene as *Rplp0* mRNA content was not altered by the treatments in vitro or in vivo. *Per1* (Mm00501813_m1), Interferon gamma (*Ifng*; Mm01168134_m1), Interleukin 18 (*Il18*; Mm00434226_m1), Interleukin 17b (*Il17b*; Mm01258783_m1), Inhibitor of Nuclear Factor Kappa B Kinase Subunit Epsilon (*Ikbke*; Mm00444862-m1), C-C Motif Chemokine Ligand 5/RANTES (*Ccl5*; Mm01302427_m1), C-C Motif Chemokine Ligand 8 (*Ccl8*; Mm01297183_m1), C-X-C motif chemokine ligand 9/MIG (*Cxcl9*: Mm00434946_m1), and C-X-C motif chemokine ligand 10/IP-10 (*Cxcl10*; Mm00445235_m1) were measured using predesigned TaqMan primer probes. The primer sequences for SYBR green reactions are shown in Table 1.

**Table 1.** SYBR Green Primer Sequences

Gene Symbol	Forward (5′-3′)	Reverse (5′-3′)	Amplicon Size, bp
*Rplp0*	CAACCCAGCTCTGGAGAAAC	GTTCTGAGCTGGCACAGTGA	169
*Per2*	AGCCACCCTGAAAAGGAAGT	GGTGAGGGACACCACACTCT	184
*Hspa1a*	TGGTGCTGACGAAGATGAAG	ATGATCCGCAGCACGTTTAG	154

### RNA Sequencing

Total RNA extracted from myotubes overexpressing *Per1, Per2*, or expressing *Gfp* within an experimental replicate was subjected to RNA sequencing. A next generation sequencing library was prepared for each sample using NEBNext Poly(A) mRNA Magnet Isolation Module followed by NEBNext Ultra II Directional RNA Library Kit for Illumina. Libraries were barcoded for multiplexing with NEBNext Multiplex Oligos for Illumina. Each multiplexed library was sequenced as a paired-end, 50 base pair sequencing run on an Illumina NovaSeq 6000, located in the Translational Science Laboratory at the Florida State University College of Medicine. Adapter trimming was performed as part of individual library demultiplexing. Quality control analysis of each library was performed using fastQC (bioinformatics.babraham.ac.uk/projects/fastqc/). Star aligner^26^ was used for mapping and alignment of the raw reads to the mouse genome (current version, GRCM39), generating counts for each gene. DESeq2^27^ was used to determine statistically significant differentially expressed genes (DEGs) in *Per1/2* overexpressing myotubes relative to *Gfp* expressing myotubes. These steps yielded lists of DEGs with a False Discovery Rate of *P *< 0.05 (adj. *P*-value) and a measure of confidence of the difference for each comparison. The raw data from the RNA sequencing analysis are publicly available in GEO (GSE299632).

### Functional Gene Enrichment and Prediction of Regulatory Transcription Factors

Upregulated and downregulated DEGs from *Per1* or *Per2* overexpressing myotubes were uploaded into the Database for Annotation, Visualization, and Integrated Discovery (DAVID; david.ncifcrf.gov) software to define the most enriched Kyoto Encyclopedia of Genes and Genomes (KEGG) pathways.^28^ DEGs were included in analyses if they had significant adjusted *P*-values ≤ 0.05 and a ± Log2 fold change ≥ 0.5. The top 500 upregulated DEGs (based upon Log2 fold change) were then uploaded into Landscape *In Silico* deletion Analysis (LISA; lisa.cistrome.org/) software to identify predicted transcription factors regulating each gene list.^29^

### Luminex Assay

Conditioned media (*n* = 5/transduction condition) was thawed on ice and cytokine/chemokine levels were measured using a custom designed Procartaplex multiplex assay (ThermoFisher) according to manufacturer instructions. Cytokine/chemokine levels in the conditioned media were determined on a Luminex Magpix system (Luminex Corporation, Austin, TX, USA) using magnetic beads and light-emitting diodes (LEDs) to detect analytes. Levels of CXCL9/MIG, CXCL10/IP-10, IFNγ, CCL5/RANTES, and IL18 were assessed using xPONENT software, in duplicate and successfully detected in all samples. No samples were excluded from the analysis. Raw fluorescence intensity data from the Luminex assay were processed using Belysa software (Belysa™ Immunoassay Curve Fitting Software, version 1.1, MilliporeSigma, Darmstadt, Germany), which enabled the generation of standard curves and the calculation of analyte concentrations through curve fitting and interpolation.

### Western Blot Analysis

Proteins were fractionated on 4-20% Tris-Glycine Criterion precast gels (Bio-Rad, Hercules, CA, USA) and transferred to polyvinylidene difluoride (PVDF) membranes. PVDF membranes were blocked in 5% non-fat dried milk in Tris-buffered saline + 0.1% Tween 20 (TBST) and membranes were incubated with primary antibodies diluted in TBST overnight at 4°C. Antibodies against Interferon Regulated Factor 3 (IRF3 Ser396; cat. #4947, diluted 1:5000), total IRF3 (cat. #4302, diluted 1:5000), Nuclear Factor Kappa Beta/Rela (NF-κB/RELA Ser536; cat. #3033, diluted 1:5000), total NF-κB/RELA (cat. #8242, diluted 1:1000), Signal Transducer and Activator of Transcription 3 (STAT3 Tyr705; cat. #9145, diluted 1:5000), total STAT3 (cat. #4904, diluted 1:5000), Extracellular Regulated Kinase 1/2 (ERK1/2 Thr202/Tyr204; cat. #9101, diluted 1:5000; cat. #2555), and total ERK1/2 (cat. #9102, diluted 1:5000; cat. #2555) were obtained from Cell Signaling Technology (Danvers, MA, USA). Primary antibodies against PER1 and PER2 were developed in the laboratory of Dr Choogon Lee. Following incubation with secondary antibodies (Anti-Rabbit IgG, Bethyl Laboratories, Montgomery, TX, USA, diluted 1:10 000, cat. #A-120-101P or Anti-Guinea Pig IgG, Sigma-Aldrich, St. Louis, MO, USA, diluted 1:10 000, cat. #A-7289), the antigen-antibody complex was visualized via enhanced chemiluminescence using Clarity reagent (cat. #1705061; Bio-Rad) on a ChemiDoc Touch imaging system (Bio-Rad). The ratio of the phosphorylated protein to the corresponding total protein was used in the analysis. Ratios were normalized to the values in the *Gfp* expressing samples.

### Statistical Analysis

Unpaired Student’s *t-*tests were used to compare normalized myotube diameter at 24 h or 48 h, gene contents between transduction conditions by qRT-PCR, and all % differences for the *Per2*+/+ and *Per2*−/− castration experiment. One-way ANOVA was used to evaluate differences in cytokine levels by Luminex and levels of phosphorylated to total protein ratios by Western blot analysis. Dunnett’s multiple comparisons test was used to assess specific pairwise comparisons when a significant F-value was observed by One-way ANOVA. Two-way ANOVA was used to evaluate muscle mass and gene expression across time in response to castration using Time and Castration as the two factors. Sidak’s multiple comparisons test was used post hoc to assess specific pairwise comparisons when an interaction was observed. All analyses were performed using GraphPad Prism Software (Version 10, La Jolla, CA, USA). Significance was set at *P* ≤ 0.05 for all analyses. All data are presented as mean ± SD or individual data points superimposed onto the mean ± SD.

## Results

### *Per2* Overexpression Induces Myotube Atrophy in Vitro While *Per2* Deletion Partially Preserves Limb Muscle Mass in Response to Androgen Deprivation

**Figure 1.**
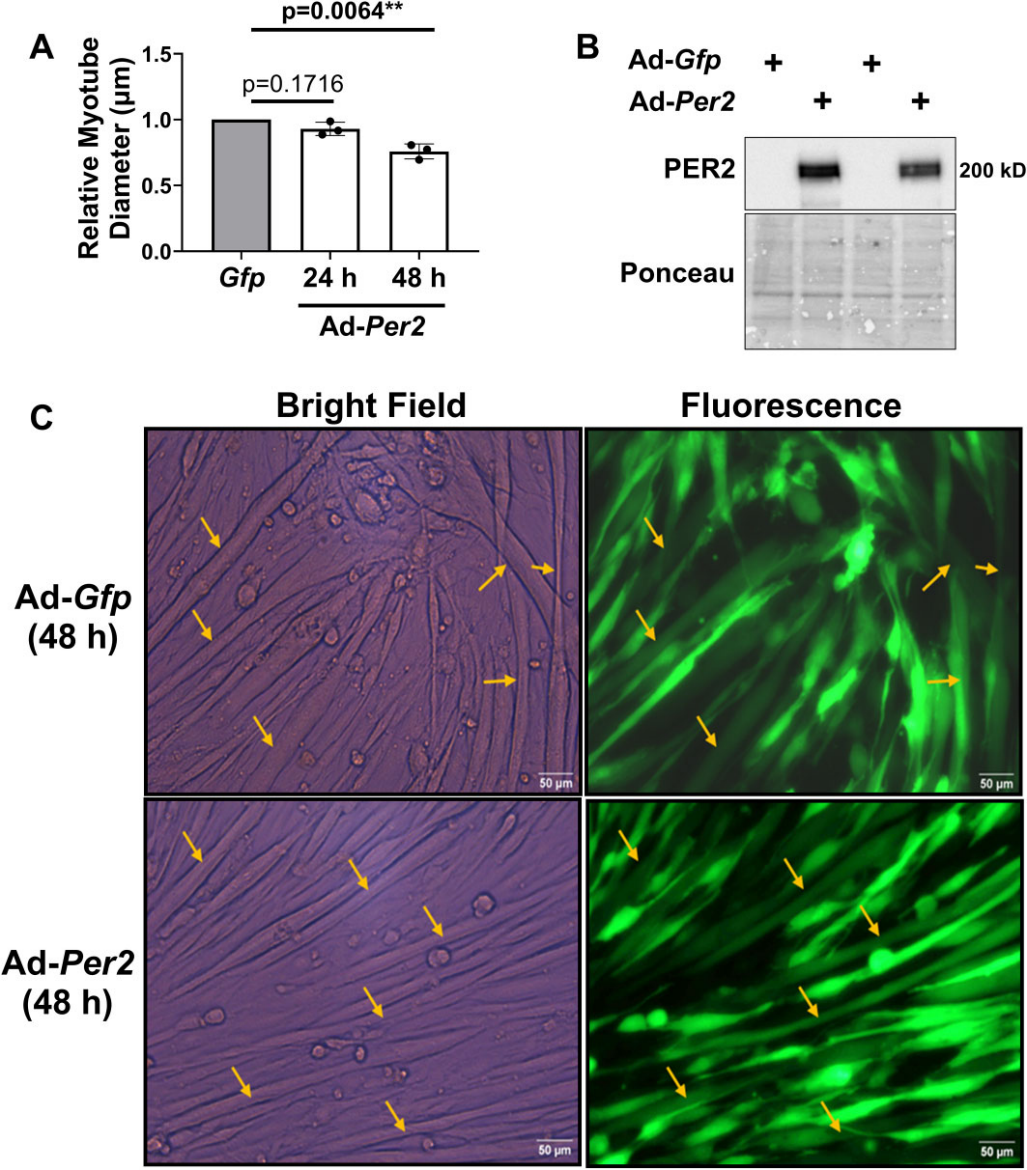
Overexpression of *Per2* induces muscle atrophy in vitro. (A) The diameters of myotubes overexpressing *Per2* relative to the diameters of myotubes overexpressing *Gfp* were determined at 24 h or 48 h post adenoviral transduction. (B) PER2 protein levels were assessed 24 h post adenoviral transduction by Western blot analysis. (C) Representative images of *Gfp* and *Per2* transduced myotubes 48 h post-transduction. Pictures are at 20×. Scalebar is 50 µm. Arrows indicate myotubes included in the analysis. All C2C12 myotube diameter experiments consisted of 3 independent experimental replicates with each replicate containing separate plates for each virus. The diameters of myotubes from each virus within a replicate were averaged to generate a single diameter value for that replicate. The mean diameter values from each replicate were used in the final analysis.

We first determined whether adenoviral-mediated overexpression of *Per2* was sufficient to induce myotube atrophy. The diameter of myotubes overexpressing *Per2* was not different than values in *Gfp* expressing myotubes 24 h post-transduction (*P *= 0.1716; Figure 1A) despite elevated PER2 protein levels in *Per2* transduced myotubes at that timepoint (Figure 1B). However, the diameter of *Per2* overexpressing myotubes was 20% thinner than *Gfp* expressing myotubes 48 h post-transduction (*P *= 0.0064; Figure 1A and C). The reduction in myotube diameter is unlikely the result of impaired myogenesis because transduction occurred only after mature myotubes had formed, and similar outcomes were observed when transduction was performed after 6 days of differentiation (data not shown).

**Figure 2.**
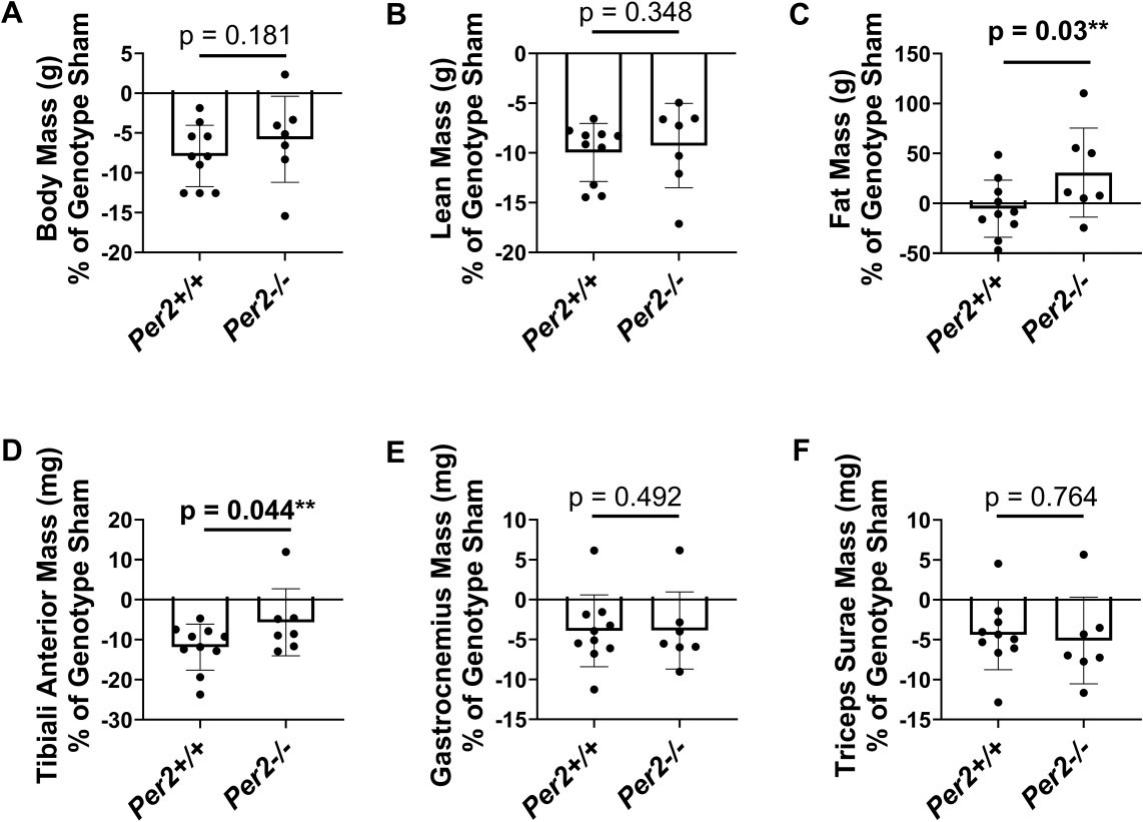
Loss of *Per2* preserves some limb muscle mass in response to androgen deprivation. (A-F) % differences in body or organ masses between the castrated group and the corresponding sham group of *Per2+/+* and *Per2−/*− mice 8 wk after a castration or sham surgery. *N* = 7-10 per group.

Since overexpression of *Per2* was sufficient to induce atrophy in vitro, we determined whether deletion of the *Per2* gene in vivo preserved limb muscle mass in response to androgen deprivation. To evaluate this, we compared the % difference between values in the castrated group to values in the sham group for each genotype, with a smaller % difference between the castrated and sham groups indicating preservation of that measure. The % differences in body mass and total lean mass between the castrated and sham groups within each genotype (*Per2+/+* or *Per2−/−*) were not different (*P* ≥ 0.181, Figure 2A-B). The % difference in fat mass was more positive in the *Per2−/*− group relative to the *Per2+/+* group (*P *= 0.03, Figure 2C), indicating fat mass was gained in response to castration in the *Per2*−/− group. The % difference in TA mass between sham and castrated groups was smaller in *Per2*−/− group compared to *Per2*+/+ group (*P *= 0.044, Figure 2D), indicating preservation of mass by *Per2* deletion. However, no genotype differences were observed for the gastrocnemius or triceps surae complex masses (*P* ≥ 0.492, Figure 2E-F). It is unlikely that loss of *Per2* altered the content of PER1 as previous work showed loss of *Per2* did not alter PER1 protein content in the liver.^30^

### *Per1* Overexpression Causes Myotube Atrophy in Vitro

**Figure 3.**
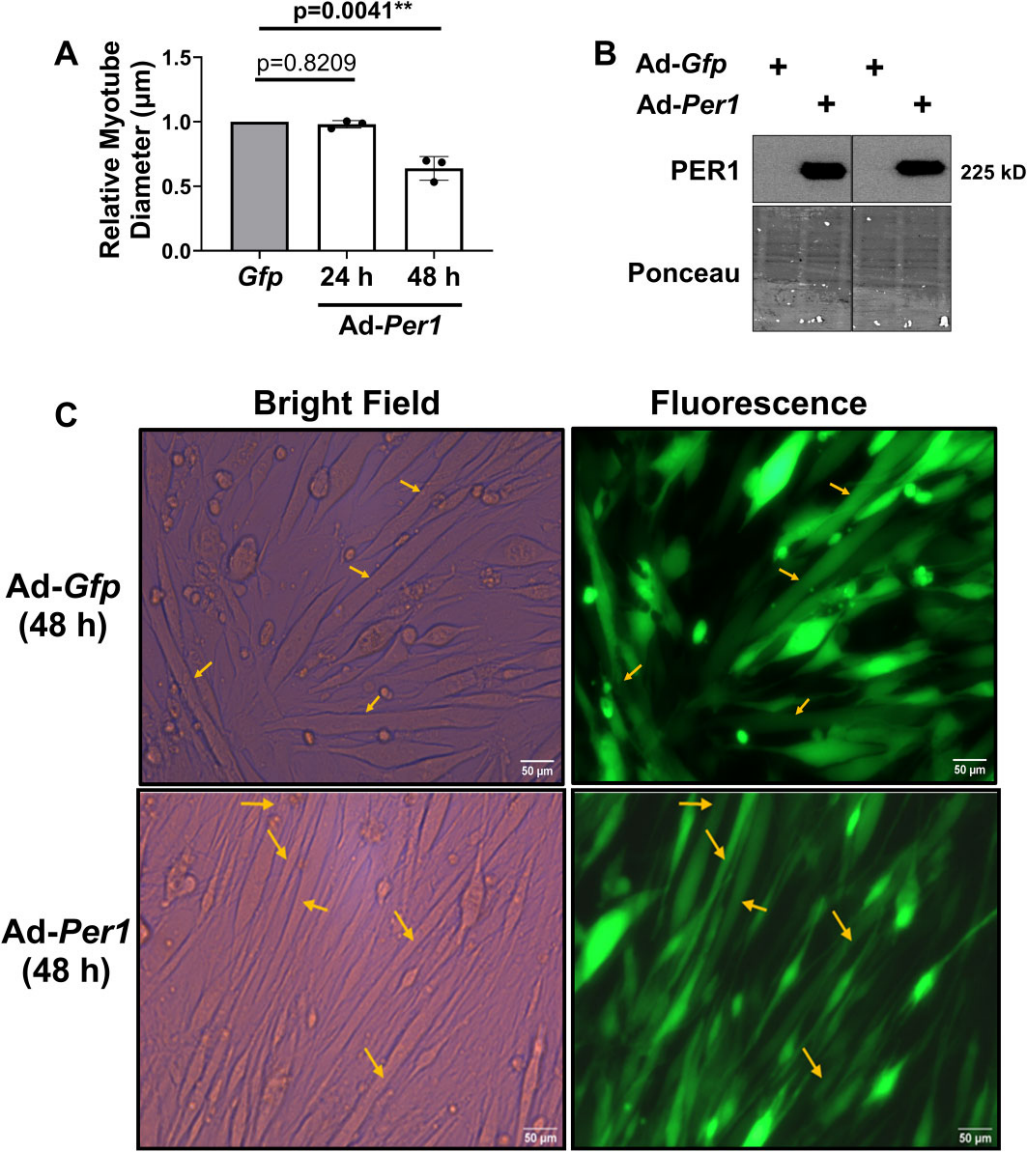
Overexpression of *Per1* induces muscle atrophy in vitro. (A) The diameters of myotubes overexpressing *Per1* relative to the diameters of myotubes overexpressing *Gfp* were determined at 24 h or 48 h post adenoviral transduction. (B) PER1 protein levels were assessed 24 h post adenoviral transduction by Western blot analysis. Line on the blot represents samples run on the same gel but in non-contiguous lanes. (C) Representative images of *Gfp* and *Per1* transduced myotubes 48 h post-transduction. Pictures are at 20×. Scalebar is 50 µm. Arrows indicate myotubes included in the analysis. All C2C12 myotube diameter experiments consisted of 3 independent experimental replicates with each replicate containing separate plates for each virus. The diameters of myotubes from each virus within a replicate were averaged to generate a single diameter value for that replicate. The mean diameter values from each replicate were used in the final analysis.

Loss of *Per2* partially maintained mass only in the TA muscle following androgen deprivation even though indices of clock disruption and mass loss occur in the gastrocnemius.^14^ Those data suggest other clock regulatory factors, such as *Per1*, may have a role in regulating muscle mass/size as PER1 in cancer cells can also function outside of the molecular clock to alter pathways that are known to regulate muscle size.^31^ The diameter of myotubes overexpressing *Per1* was not different than *Gfp* expressing myotubes 24 h post-transduction (*P *= 0.8209; Figure 3A) despite elevated PER1 protein levels in *Per1* transduced myotubes at that timepoint (Figure 3B). However, at 48 h post-transduction, myotube diameter was 31% thinner in *Per1* overexpressing myotubes relative to *Gfp* expressing myotubes (*P *= 0.0064; Figure 3A and C). We were unable to co-transduce myotubes with *Per1* and *Per2* as the viral load required for both viruses to fully transduce myotubes led to myotube death.

### *Per1* and *Per2* Overexpression Induces a Proinflammatory Gene Expression Signature in C2C12 Myotubes

**Figure 4.**
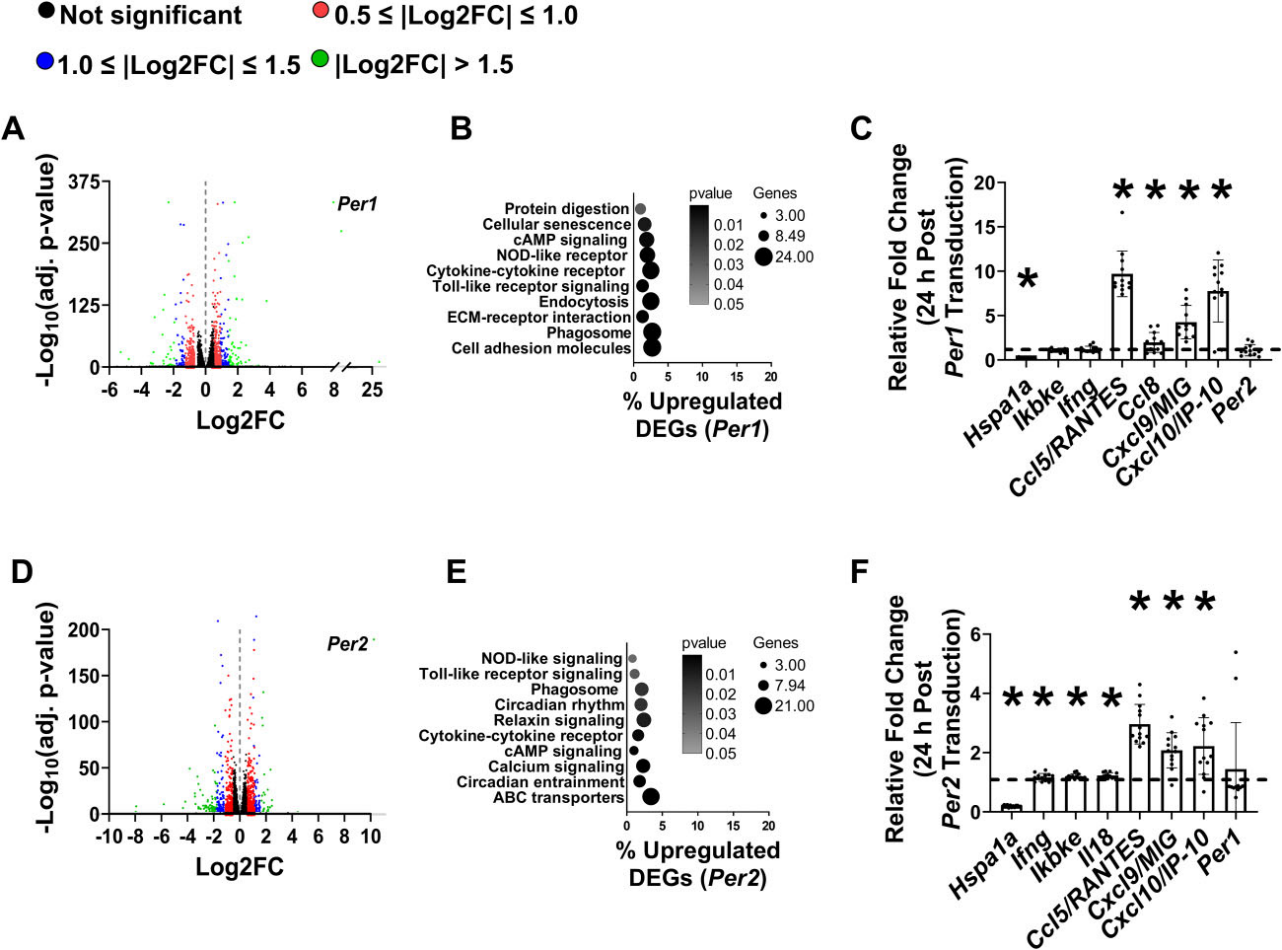
Overexpression of *Per1* or *Per2* induces an inflammatory gene expression signature in myotubes. (A) Volcano plot illustrating −Log_10_ fold changes (FC) of genes from C2C12 myotubes overexpressing *Per1* relative to myotubes expressing *Gfp* following RNA sequencing. Labeled dot represents *Per1* mRNA data point. (B) Enriched KEGG pathways from upregulated differentially expressed genes (DEGs) from C2C12 myotubes overexpressing *Per1* relative to myotubes expressing *Gfp*. (C) The mRNA contents of heat shock protein 70 (*Hspa1a*), inhibitor of nuclear factor kappa B kinase subunit epsilon (*Ikbke*), interferon gamma (*Ifng*), C-C motif chemokine ligand 5 (*Ccl5/*RANTES), C-C motif chemokine ligand 8 (*Ccl8*), C-X-C motif ligand 9 (*Cxcl9*/MIG), C-X-C motif ligand 10 (*Cxcl10*/IP-10), and *Per2* were measured in myotubes 24 h after transduction with adenovirus overexpressing *Per1* and made relative to values in myotubes transduced with adenovirus expressing *Gfp. N* = 11-13 per group from 3 independent experiments. (D) Volcano plot illustrating −Log_10_ FC of genes from C2C12 myotubes overexpressing *Per2* relative to myotubes expressing *Gfp* following RNA sequencing. Labeled dot represents *Per2* mRNA data point. (E) Enriched KEGG pathways from upregulated DEGs from C2C12 myotubes overexpressing *Per2* relative to myotubes expressing *Gfp*. (C) The mRNA contents of *Hspa1a, Ikbke, Ifng, Ccl5*/RANTES, *Ccl8, Cxcl9*/MIG, *Cxcl10*/IP-10, and *Per1* were measured in myotubes 24 h after transduction with adenovirus overexpressing *Per2* and made relative to values in myotubes transduced with adenovirus expressing *Gfp. N* = 11-13 per group from 3 independent experiments. *Significantly different than values in myotubes expressing *Gfp* at *P* < 0.05.

PER1 and PER2 can function outside the core clock to alter activity of pathways that regulate gene expression.^21^^,^^32^ To investigate potential transcriptional mechanisms underlying myotube atrophy following *Per1* or *Per2* overexpression, we performed bulk RNA sequencing on myotubes 48 h post-transduction. RNA sequencing identified 1592 differentially expressed genes (DEGs) from myotubes overexpressing *Per1* comprising 616 upregulated (including *Per1*) and 976 downregulated DEGS (Figure 4A and Supplemental Table S1). KEGG pathway analysis of all DEGs revealed Cytokine-cytokine receptor interaction as the most enriched pathway, suggesting *Per1* caused a prominent inflammatory gene expression signature. When the KEGG pathway analysis was repeated on only the upregulated DEGs to increase pathway enrichment,^33^ inflammatory pathway enrichment became more prominent and included NOD-like receptor, Cytokine-cytokine receptor, and Toll-like receptor signaling (Figure 4B). Notably, the genes induced by *Per1* overexpression did not include putative atrophy associated genes such as Muscle Ring Finger 1 (*Murf1*) or Muscle Atrophy F-box (*Mafbx*), nor did it include pathways shown to be altered by *Per1* induction in cancer cells. The top transcription factor predicted to influence the upregulated gene set was Signal Transducer and Activator of Transcription 2 (STAT2; Table 2), a factor closely linked to inflammatory signaling.^34^

**Table 2.** Transcription Factors Predicted to Regulate the Upregulated Genes 48 h Following *Per1* Induction

Transcription Factor (Upregulated Genes)	−Log_10_ *P*-value
STAT2	28.94
ERG	14.01
RUNX2	13.87
TRIM63	13.68
RARA	12.87

STAT2; Signal Transducer and Activator of Transcription 2, ERG; ETS Transcription Factor ERG, RUNX2; Runt-Related Transcription Factor 2, TRIM63; Tripartite Motif Containing 63 (aka Muscle Specific RING Finger Protein 1), RARA; Retinoic Acid Receptor Alpha.

Although myotube atrophy was not present 24 h post-transduction, the content of select inflammatory associated/inflammation regulatory genes identified by the RNA sequencing with known roles in muscle size regulation^35^^,^^36^^,^^37^^,^^38^^,^^39^^,^^40^ were still modestly to largely altered 24 h post-transduction. Specifically, the mRNA content of heat shock protein 70 (*Hspa1a*) was lower while contents of *Ccl8, Ccl5/*RANTES, *Cxcl9*/MIG, and *Cxcl10*/IP-10 were modestly to substantially higher 24 h following *Per1* induction (*P* ≤ 0.0073, Figure 4C). The mRNA contents of *Ikbke* and *Ifng* were not different between conditions at the 24 h post-transduction time point (*P* ≥ 0.076; Figure 4C) despite elevation at the 48 h post-transduction timepoint. *Per1* induction did not alter *Per2* mRNA content at the 24 h time point (Figure 4C).

Similar to *Per1* overexpression, *Per2* overexpression also altered the muscle transcriptional profile. RNA sequencing identified 1448 DEGs from myotubes overexpressing *Per2* comprising 665 upregulated (including *Per2*) and 783 downregulated DEGs (Figure 4D and Supplemental Table S2). Similar to *Per1*, Cytokine-cytokine receptor interaction was the top KEGG pathway enriched when all DEGs were analyzed. Analysis of only the upregulated DEGs from *Per2* overexpressing myotubes again highlighted enrichment into several inflammatory related pathways such as NOD-like signaling, Toll-like receptor signaling, Cytokine-cytokine signaling, and ABC transporters (Figure 4E). Also consistent with *Per1*, the genes induced by *Per2* overexpression did not include putative atrophy associated genes such as *Murf1* or *Mafbx* or those shown to be altered by *Per2* induction in cancer cells. The top transcription factors predicted to influence the upregulated gene set also included the inflammatory associated STAT transcription factors (Table 3).

**Table 3.** Transcription Factors Predicted to Regulate the Upregulated Genes 48 h Following *Per2* Induction

Transcription Factor (Upregulated Genes)	−Log_10_ *P*-value
STAT2	34.98
STAT1	23.13
TRIM63	19.44
MECP2	11.55
IRF4	10.11

STAT2; Signal Transducer and Activator of Transcription 2, STAT1; Signal Transducer and Activator of Transcription 1, TRIM63; Tripartite Motif Containing 63 (aka Muscle Specific RING Finger Protein 1), MECP2; Methyl-CpG Binding Protein 2, IRF4; Interferon Regulatory Factor 4.

Although myotube atrophy was not present 24 h post-transduction, the content of select inflammatory-associated genes identified by the RNA sequencing with known roles in muscle size regulation^35^^,^^36^^,^^37^^,^^38^^,^^39^^,^^40^ were still modestly to largely altered 24 h post-transduction. The mRNA content of *Hspa1a* was lower while contents of *Ifng, Ikbke, Il18, Ccl8, Ccl5*/RANTES, *Cxcl9*/MIG, and *Cxcl10*/IP-10 were modestly to moderately higher 24 h following *Per2* induction (*P* ≤ 0.0073, Figure 4F). *Per2* induction did not alter *Per1* mRNA content at the 24 h time point (Figure 4F)

### Autocrine-Mediated Signals Contribute to *Per1*- and *Per2*-Induced Myotube Atrophy

**Figure 5.**
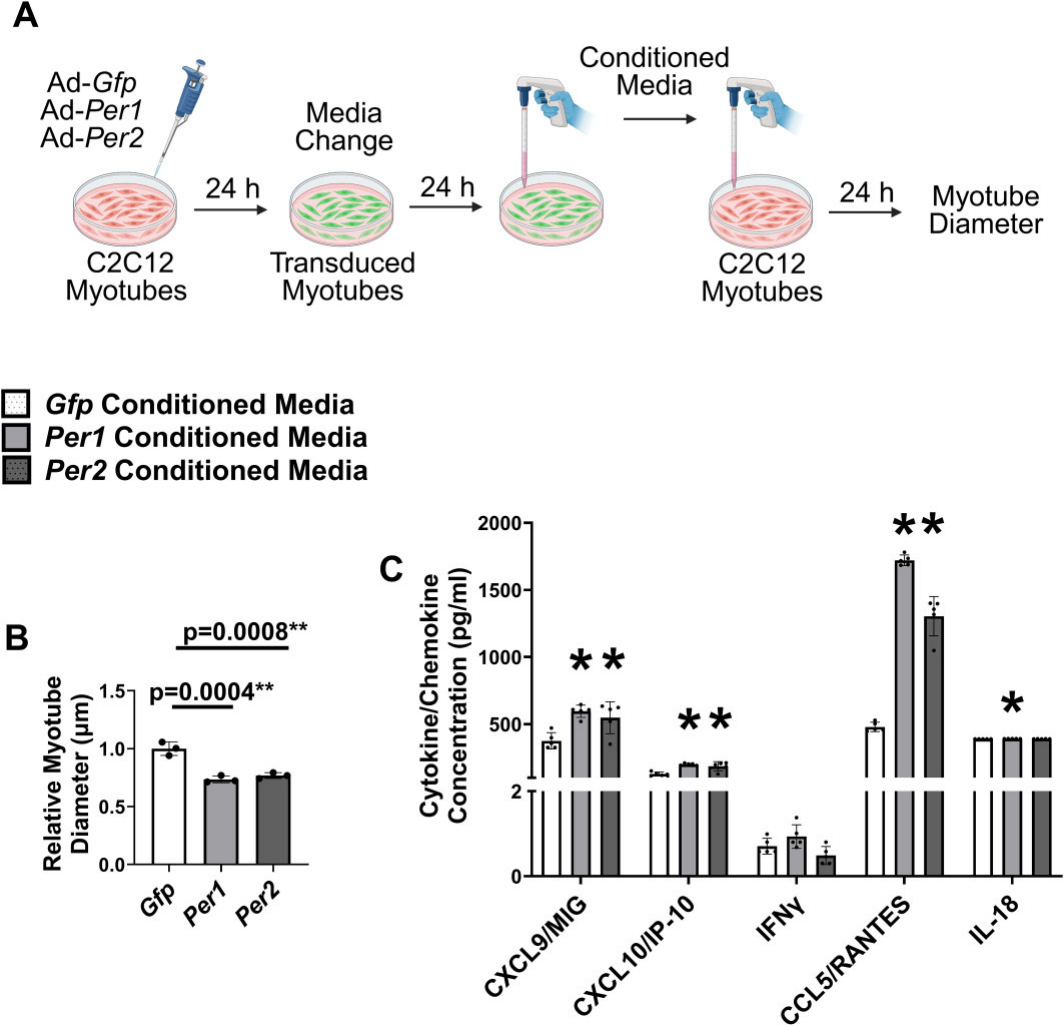
*Per1* or *Per2* overexpression induce myotube atrophy via an autocrine-mediated mechanism. (A) Experimental design for conditioned media experiment. (B) Diameter of C2C12 myotubes incubated for 24 h in media conditioned from C2C12 myotubes transduced with adenovirus overexpressing *Per1, Per2*, or *Gfp*. (C) Concentrations of C-X-C motif ligand 9 (CCL9/MIG), C-X-C motif ligand 10 (CXCL10/IP-10), Interferon Gamma (IFNγ), CCL5/RANTES, and IL18 in the media conditioned from C2C12 myotubes transduced with adenovirus overexpressing *Per1, Per2*, or *Gfp. N* = 5 per transduction condition. *Significantly different than *Gfp* conditioned media at *P* < 0.05.

Inflammatory cytokines and chemokines regulate muscle size in part by surface receptor signaling cascades.^41^ Given the inflammatory gene expression profiles induced by *Per1* or *Per2* overexpression (specifically chemokines), we hypothesized the myotube atrophy induced by *Per1* or *Per2* overexpression was driven in part by an autocrine-mediated process. To test this, mature C2C12 myotubes were incubated for 24 h in the media conditioned by myotubes overexpressing *Gfp, Per1*, or *Per2* (Figure 5A). Exposure to *Per1* or *Per2* conditioned media for 24 h lowered myotube diameter by 24% and 21%, respectively, relative to the diameter of myotubes incubated in *Gfp* conditioned media (Figure 5B). Consistent with an autocrine-mediated mechanism involving secreted inflammatory factors, concentrations of select cytokines/chemokines identified by the RNA sequencing and which are associated with muscle size regulation^42^^,^^43^ including CXCL9/MIG, CXCL10/IP-10, and CCL5/RANTES were higher in the *Per1* and *Per2* conditioned media compared to *Gfp* conditioned media (Figure 5C). Concentrations of IL18 were slightly higher only in the *Per1* conditioned media relative to *Gfp* conditioned media, although the increase was minimal (389.9 ± 0.41 vs. 391.0 ± 0.53; Figure 5C). Glucose levels in the *Gfp, Per1*, and *Per2* conditioned medias prior to treating non-transduced myotubes were 549, 494, and 507 mg/dL, respectively. The amount of glucose used by the non-transduced myotubes during the incubation did not differ between groups (Δ132.7 ± 8.5, Δ116 ± 17.7, and Δ121.3 ± 6.5 mg/dL for *Gfp, Per1*, and *Per2*, respectively), although the numeric values for consumption were lower in the myotubes incubated in the *Per1* and *Per2* conditioned media. The pH of the conditioned medias after incubating the non-transduced myotubes were all ∼7.5.

### *Per1* and *Per2* Overexpression Leads to Increased Phosphorylation of the Interferon Regulated Factor 3 (IRF3) Transcription Factor

**Figure 6.**
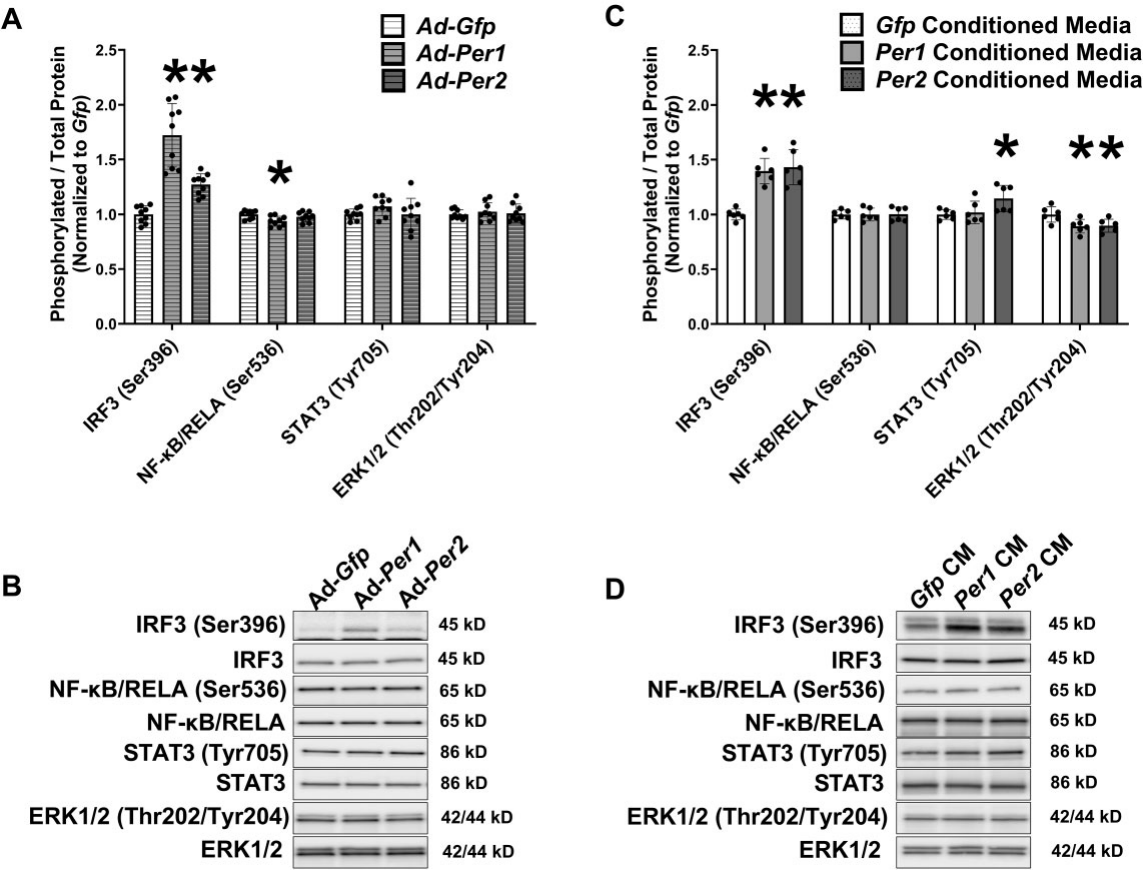
*Per1* and *Per2* overexpression leads to increased phosphorylation of the Interferon Regulated Factor 3 (IRF3) transcription factor. (A) The phosphorylated to total protein ratios for IRF3 (Ser396), Nuclear factor kappa beta/RELA (NF-κB/RELA; Ser536), Signal transducer and activator of transcription 3 (STAT3; Tyr705), and Extracellular signal regulated kinase1/2 (ERK1/2; Thr202/Tyr204) in C2C12 myotubes 24 h after transduction with adenovirus overexpressing *Per1, Per2*, or *Gfp. N* = 9 per transduction condition from 3 independent experiments. (B) Representative Western blot. (C) The phosphorylated to total protein ratios for IRF3 (Ser396), NF-κB/RELA (Ser536), STAT3 (Tyr705), and ERK1/2 (Thr202/Tyr204) in C2C12 myotubes 1 h after exposure to media conditioned from C2C12 myotubes transduced with adenovirus overexpressing *Per1, Per2*, or *Gfp* for 48 h. *N* = 6 per condition from 3 independent experiments. (D) Representative Western blot. *Significantly different than either Ad-*Gfp* or *Gfp* CM at *P* < 0.05.

Although several inflammatory associated genes were upregulated prior to myotube atrophy in response to *Per1* or *Per2* overexpression, the contents of *Ccl5*/RANTES, *Cxcl9*/MIG, and *Cxcl10*/IP-10 were induced to higher levels (Figure 4C and F). Those chemokines are known targets of transcription factors including IRF3,^44^ and accordingly, phosphorylation of IRF3 on the Ser396 activation site^45^ was higher in both *Per1* and *Per2* overexpressing myotubes 24 h post-transduction (*P* ≤ 0.0079, Figure 6A-B). Those chemokines are also transcriptional targets of Nuclear Factor Kappa Beta (NF-κB) signaling,^46^ with phosphorylation of NF-κB/RELA indicating increased transcriptional activity.^47^ However, NF-κB/RELA (Ser536) phosphorylation was modestly lower in *Per1* overexpressing myotubes 24 h post-transduction (*P *= 0.0077) while phosphorylation was not altered at that time point in *Per2* overexpressing myotubes (Figure 6A-B). Consistent with a lack of NF-κB/RELA transcriptional induction, protein content of Inhibitor of Kappa Beta alpha, which binds to and inhibits NF-κB nuclear translocation and is degraded upon activation,^48^ was not different between groups 24 h post-transduction (data not shown). Although chemokines can increase or suppress phosphorylation of other inflammatory associated signaling proteins including STAT3 and ERK1/2, respectively, via membrane receptor signals,^49^^,^^50^ phosphorylation of neither protein was different between groups (Figure 6A-B), suggesting autocrine-mediated inflammatory signaling was not yet prominent at the 24 h post-transduction time point.

Because the inflammatory gene expression profile was more pronounced at 48 h post-transduction, and *Per1* or *Per2* conditioned medias collected at that time point induced myotube atrophy, we assessed whether exposing myotubes to those conditioned medias was sufficient to also increase IRF3 phosphorylation and alter other inflammatory-sensitive pathways. Myotubes incubated for 1 h in *Per1* or *Per2* conditioned media exhibited higher phosphorylation of IRF3 (Ser396) relative to myotubes exposed to *Gfp* conditioned media (Figure 6C-D). Phosphorylation of NF-κB/RELA (Ser536) was again not different between conditions while STAT3 (Tyr705) phosphorylation was modestly elevated in the myotubes acutely exposed to *Per2* conditioned media (Figure 6C-D). Phosphorylation of ERK1/2 (Thr202/Tyr204) was modestly lower in myotubes incubated in either *Per1* or *Per2* conditioned media (Figure 6C-D).

### A Proinflammatory Gene Expression Signature Coincides With the Loss of Limb Muscle Mass in Response to Androgen Deprivation

**Figure 7.**
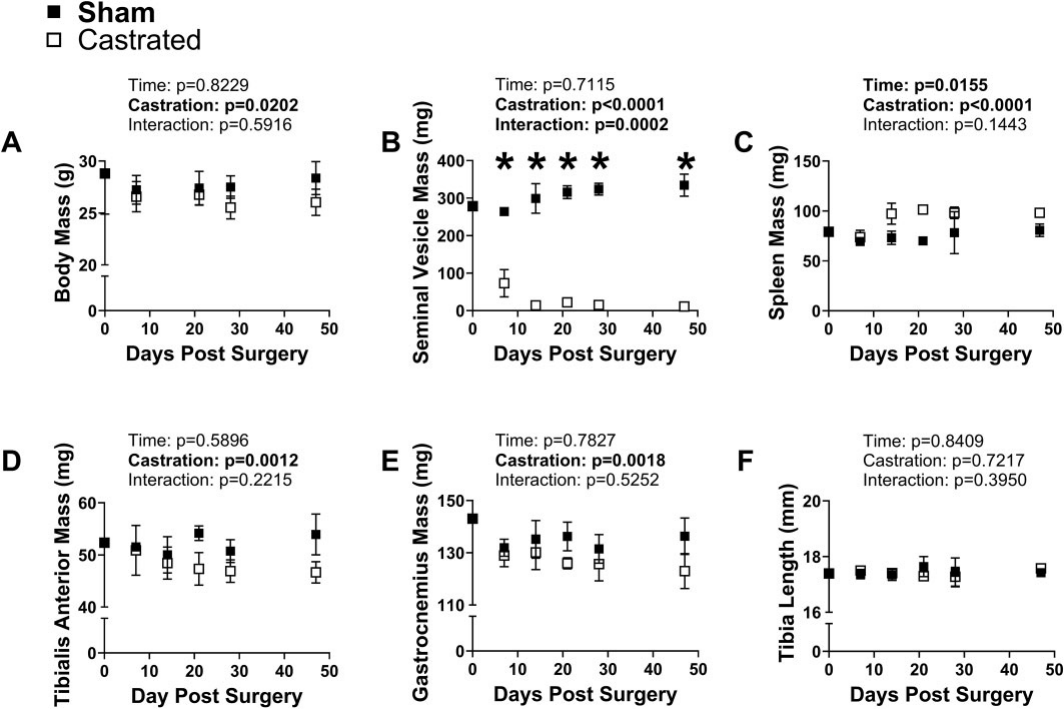
Time course of tissue characteristics following castration. (A) body mass, (B) seminal vesicle (SV) mass, (C) spleen mass, (D) tibialis anterior mass, (E) gastrocnemius mass, and (F) tibia length following castration or sham surgery. *Significantly different via post hoc testing. Otherwise, main effects are reported. *N* = 3-4 per group at each time point.

Our in vitro analyses suggest the regulation of muscle size by *Per1* or *Per2* accumulation is mediated in part by inflammatory signaling. To evaluate whether contents of the inflammatory genes identified following *Per1* or *Per2* overexpression in vitro were altered in limb muscle in response to androgen deprivation, and to evaluate when in the progression of limb muscle mass loss those changes were most prominent, we performed a time course analysis following castration. As expected, body mass and seminal vesicle mass values were lower in response to castration (Castration: *P* ≤ 0.0202, Interaction: *P *= 0.0002) while spleen mass was higher in the castrated groups (Castration: *P *< 0.0001, Figure 7A-C). TA and gastrocnemius muscle masses were also lower in the castrated groups compared to sham values (Castration: *P* ≤ 0.0018, Figure 7D-E), particularly at the 49-day time point. Those muscle mass differences between groups were not due to changes in body size as tibia length was not different between groups (Castration: *P *= 0.7217) nor did tibia length change across the time course (Time: *P *= 0.8409, Figure 7F). Notably, both body mass and limb muscle mass values in the sham groups were relatively stable across the time course with the values at day 49 post-surgery being similar to the values obtained on the day of the surgery (day 0), indicating a steady state had been reached prior to starting the experiment.

**Figure 8.**
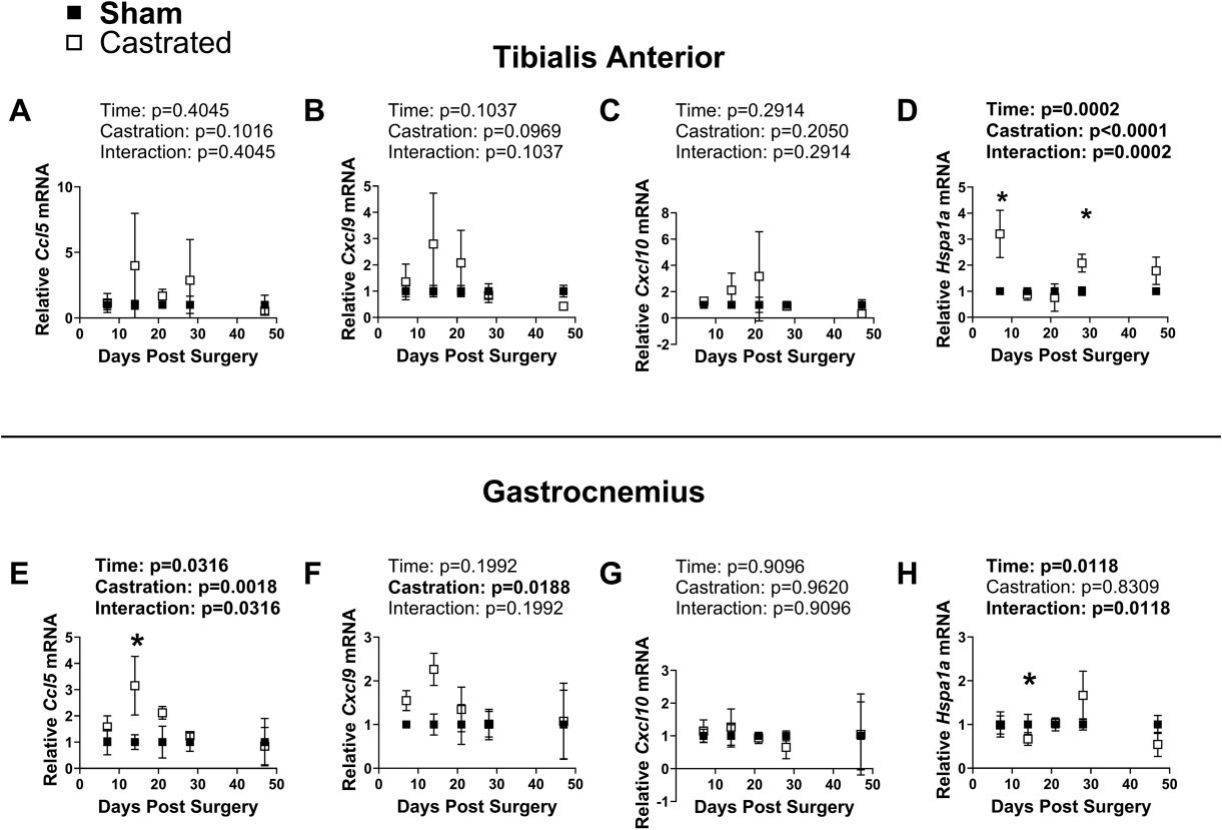
Time course of inflammatory associated genes following castration. The mRNA contents of C-C motif chemokine ligand 5 (*Ccl5*/RANTES), C-X-C motif ligand 9 (*Cxcl9*/MIG), C-X-C motif ligand 10 (*Cxcl10*/IP-10), and Heat shock protein 70 (*Hspa1a*) were determined in the (A-D) tibialis anterior muscle or (E-H) the gastrocnemius muscle by qRT-PCR. *Significantly different via post hoc testing. Otherwise, main effects are reported. *N* = 3 per group at each time point.

The mRNA contents of cytokines induced by *Per1* or *Per2* overexpression in vitro including *Ifng, Ikbke, Il17b, and Il18* were below detectable levels in all TA and gastrocnemius muscle samples. In the TA, *Ccl5*/RANTES, *Cxcl9*/MIG, and *Cxcl10*/IP-10 mRNA contents were numerically, but not significantly, higher in the castrated groups, especially during the early time points post-surgery (Castration: *P* ≥ 0.0969; Figure 8A-C).The mRNA content of *Hspa1a* was overall higher in the TA of the castrated groups throughout the time course (Castration: *P *< 0.0001) except for the 14- and 21-day post-surgery time points (Interaction: *P *= 0.0002) where values were not different between groups (Figure 8D). In the gastrocnemius, *Ccl5*/RANTES and *Cxcl9*/MIG mRNA contents were significantly higher in the castrated groups (Castration: *P *= 0.0018), especially during the early post-surgery time points (Figure 8E-F). *Cxcl10*/IP-10 mRNA content in the gastrocnemius was not different between groups (Castration: *P *= 0.9620; Figure 8G). *Hspa1a* mRNA content was lower in the gastrocnemius of the castrated group at the 14-day post-surgical timepoint (Interaction: *P* = 0.0118; Figure 8H).

## Discussion

Loss of limb muscle mass is a primary discernable feature in men with hypogonadism.^4^ The molecular clock has recently emerged as an important regulator of skeletal muscle health,^16^ and our current work shows increased expression of the primary clock suppressor, *Per2*, is sufficient to promote muscle atrophy in vitro. Moreover, *Per2* deletion in vivo partially maintains some limb muscle mass in response to low androgen levels. We extend that work to show increased expression of the other primary molecular clock suppressor, *Per1*, also induces muscle atrophy in vitro. The *Per1/2* mediated regulation of muscle size occurs in part through an autocrine-mediated process that likely involves inflammation with several of the inflammatory genes identified in vitro being altered during the early stages of limb muscle mass loss in response to low androgen levels. Overall, these data identify *Per1* and *Per2* as novel atrophy-inducing genes with direct implications for their roles in regulating limb muscle mass loss during hypogonadism.

Our finding that sustained *Per1 or Per2* induction promotes muscle atrophy likely by inducing an inflammatory gene expression signature is consistent with work in other cell types showing the PERs modulate inflammation. For example, disrupting *Per2* in macrophages lowered the release of TNFα and IL-12 following stimulation of Toll Like Receptor 9^51^ while loss of *Per1* upregulated immune response related genes in induced neuron cells in culture.^52^ However, the inflammation induced by *Per1 or Per2* overexpression in muscle likely differs from those other cell types as it occurred intrinsically in myotubes without the presence of immune cells or inflammatory-inducing agents (eg, LPS). Our data point to IRF3 as a likely mediator of the initial inflammatory gene expression response driven by *Per1* or *Per2* overexpression, as IRF3 phosphorylation was higher in *Per1/2* overexpressing myotubes prior to the myotube atrophy (24 h post-transduction). Subsequent changes to the inflammatory gene signature following longer term *Per1* or *Per2* overexpression were then likely mediated by sustained IRF3 activation combined with the addition of other inflammatory sensitive signaling cascades. In support of this conclusion, conditioned media generated from myotubes overexpressing *Per1* or *Per2* for 48 h increased IRF3 phosphorylation and altered phosphorylation of other signaling proteins responsive to inflammatory mediators (eg, STAT3) that were not altered by *Per1* or *Per2* induction at the 24 h time point. While direct activation of IRF3 via *Per1* or *Per2* has not been demonstrated to our knowledge, we speculate IRF3 activation likely occurred in part by signals such as genomic instability. Prior work showed low androgens make cells susceptible to DNA damage by downregulating DNA repair genes,^53^ and subsequent release of DNA into the cytosol can trigger IRF3 activation.^54^ Thus, PER1/2-mediated IRF3 activation in muscle may reflect a broader interaction between molecular clock regulators, stress responses, and innate immune signaling.

Hypogonadism is linked to systemic inflammation,^55^^,^^56^ yet there is only one known report where inflammatory gene expression was assessed in muscle when androgen levels were low. Jiao et al.^57^ showed mRNA contents of tumor necrosis factor alpha (*Tnfα*), interleukin 1 alpha (*Il1α*), and interleukin 6 (*Il6*) were not altered in the atrophied gastrocnemius of castrated rats compared to values in the gastrocnemius of uncastrated rats, suggesting inflammation is not a predominant regulator of limb muscle mass following androgen deprivation (at least at later time points post-deprivation). However, our data indicate the *Per2*-mediated regulation of limb muscle mass in response to androgen deprivation (ie, Figure 2), and potentially *Per1*, likely involves a set of inflammatory mediators distinct from those reported by Jiao et. al. Indeed, *Tnfα* and *Il1α* mRNA contents were not altered in myotubes overexpressing *Per1* or *Per2* while *Il6* mRNA content was lower following *Per1* or *Per2* inductions. Rather, our in vitro data suggest chemokines such as CXCL9/MIG, CXCL10/IP-10, and CCL5/RANTES are likely more predominant drivers of the atrophy following *Per1/2* inductions as levels of those chemokines were elevated by a greater magnitude in the *Per1/2* conditioned medias compared to cytokines. Possible chemokine driven atrophy in vivo is likely to be most prominent during the initial stages of muscle mass loss in response to low androgen levels. In the gastrocnemius, *Ccl5*/RANTES and *Cxcl9*/MIG mRNA contents were elevated by the greatest magnitudes during the initial time points following androgen deprivation. While similar trends for those two chemokines were also observed in the TA, statistical significance was not reached due to limited power at the current sample size. Moreover, Heat Shock Protein 70 can have immunosuppressive actions,^58^ and *Hspa1a* mRNA was not elevated in the TA at the early post-surgical time points when *Ccl5/Cxcl9* mRNAs were numerically higher while *Hspa1a* levels were lower in the gastrocnemius at those early post-surgical time points. Together, our data suggest PER2 and possibly PER1 induce limb muscle mass loss in response to low androgen levels likely by promoting a low-grade inflammatory state that is prominent during the initial stages of muscle mass loss.

The muscle phenotype and corresponding inflammatory gene expression signature following *Per1/2* inductions contrast the only other known report where *Per1/2* were overexpressed in muscle cells. In that report, *Per1/2* levels were increased in proliferating C2C12 myoblasts, resulting in enhanced rates of differentiation by increasing expression of genes such as Insulin like growth factor 2 (*Igf2*) and Myogenin (*Myog*).^59^ Our RNA sequencing analyses did not find induction of either *Igf2* or *Myog* when *Per1* or *Per2* were overexpressed in fully differentiated myotubes. The contrasting data could be due to the timing of *Per1/2* overexpression (myoblasts vs. fully differentiated myotubes) or that the control group in our study overexpressed a protein (GFP) rather than transduction with an empty vector. Regardless, it appears overexpression of *Per1/2* in fully developed myotubes leads to distinctly different gene expression profiles and subsequent phenotypes compared to when *Per1/2* are induced in undifferentiated myocytes.

While our work connects androgen deprivation to muscle atrophy via the molecular clock, links between androgens and other circadian behaviors/physiological processes have been established. For instance, testosterone alters circadian running wheel activity in young and aged male rodents.^60^ Likewise, physiological processes with circadian patterns such as sleep and blood pressure are also controlled in part by testosterone.^61^^,^^62^^,^^63^ Those effects of androgens on circadian behavior/physiology may be mediated in part by the molecular clock as genetic disruption of the clock leads to similar changes to those behaviors/physiological processes.^64^^,^^65^^,^^66^ Thus, the current findings extend upon the breadth of the androgen-circadian literature to include muscle mass regulation.

Overall, our data show overexpression of the primary molecular clock suppressors *Per1* and *Per2* promote muscle atrophy through an autocrine-mediated process likely involving inflammation with implications for their role in promoting muscle mass loss in response to low androgen levels. Despite these novel findings, several important limitations and unanswered questions remain and warrant future investigation. First, loss of *Per2* in muscle alters body temperature, total locomotor activity, and expression of proteins related to metabolism.^67^ Thus, the total loss of PER2 in the current study may have broader systemic effects when combined with androgen deprivation. Second, the role of *Per1* in the promotion of limb muscle mass loss in response to androgen deprivation requires loss of function experiments. Third, whether a more modest elevation of *Per1* and *Per2* elicits a similar inflammatory gene expression profile and muscle atrophy is unknown. Fourth, our work studied the effect of sustained changes to the *Per1/2* genes, and thus, future work should assess how daily *Per1/2* oscillations impact muscle mass in response to androgen deprivation. Fifth, the mechanism(s) by which *Per1* and *Per2* promote an intramuscular inflammatory gene expression profile and the extent to which inflammation contributes to the muscle atrophy are also ill defined. Addressing these limitations/unanswered questions in future work will provide novel insight into the androgen regulation of limb muscle mass as well as the novel role for *Per1/2* to the regulation of skeletal muscle physiology.

## Supplementary Material

zqaf030_Supplementary_Data

## Data Availability

The data that support the findings of this study are available at GEO or are available from the corresponding author upon reasonable request.
