# Supplementary material for: Sustained Accumulation of Molecular Clock Suppressors Period 1 and Period 2 Promotes C2C12 Myotube Atrophy Through an Autocrine-Mediated Mechanism With Relevance to Androgen Deprivation-Induced Limb Muscle Mass Loss
Source: Function (Oxf). 2025 Jul 9;6(4):zqaf030. doi: 10.1093/function/zqaf030 (PMC12316099; doi:10.1093/function/zqaf030)
Supplement: zqaf030_Supplementary_Data [file zqaf030_supplementary_data.zip › Additional Supplementary data.docx]

**Additional Supplementary data**

**These Supplementary data files do not form part of the PDF but are available from the Function Editorial Office (**function.editorialoffice@paeditorial.co.uk**) on request:**

1. Supplemental Tables 1 and 2.xlsx
